# Editorial: Prof. Frank Rösch's legacy in the radiopharmaceutical chemistry field

**DOI:** 10.3389/fnume.2023.1336481

**Published:** 2023-11-27

**Authors:** Christian B. M. Poulie, Markus Piel, Bernd Neumaier, Tobias Ross, Matthias Herth

**Affiliations:** ^1^ Department of Drug Design and Pharmacology, Faculty of Health and Medical Sciences, University of Copenhagen, Copenhagen, Denmark; ^2^ Department of Chemistry, Johannes Gutenberg-Universität Mainz, Mainz, Germany; ^3^ Department of Chemistry, Faculty of Chemistry, Pharmaceutical Sciences, Geography and Geosciences, Johannes Gutenberg-University Mainz, Mainz, Germany; ^4^ Department of Nuclear Medicine, Hannover Medical School, Hannover, Germany; ^5^ Department of Clinical Physiology, Nuclear Medicine & PET, Copenhagen University Hospital, Rigshospitalet, Copenhagen, Denmark

**Keywords:** radiopharmaceutical chemistry, nuclear chemistry/radiochemistry, radiometal, fluorine-18, gallium 68

**Editorial on the Research Topic** Prof. Frank Rösch’s legacy in the radiopharmaceutical chemistry field

Recent years have witnessed a remarkable acceleration in radiopharmaceutical research and development. The further integration of radiochemistry and nuclear medicine into molecular biology has led to an era of precision diagnostics and targeted therapies. Tailoring treatments to individual patients and their unique disease characteristics has become a reality. This not only increases the effectiveness of therapies but also minimizes side effects, improving the overall quality of patient care. The advent of theranostics, which combines diagnostics and therapeutics, exemplifies this paradigm shift, and it is an area where radiopharmaceutical sciences will be playing a pivotal role.

This Research Topic is dedicated to Prof. Dr. Frank Rösch, who has contributed an immense amount of groundbreaking research to the field over his career. Not only he has pushed the limits within our field especially within the theranostic field, but also educated a vast number of radiopharmaceutical scientists that are now located around the globe. He supervised about 60 PhD and numerous Diploma, Master and BSc students, and many postdoctoral fellows and visiting scientists. The scientific work of Frank Rösch, documented in about 400 publications and 30 national and international patents, shows him as a scientist of extraordinary versatility, breadth and depth. He contributed many chapters in important scientific books, wrote and edited teaching books for students and professionals and was a key person in creating the Handbook on Nuclear Chemistry, a 3,600 pages book series presenting the knowledge on radio and nuclear chemistry.

Frank Rösch was also honored with several prizes, starting with Scientific Awards from the Technical University Dresden and the Central Research Institute of the Academy of Sciences Rossendorf in his early career and later on twice with the Prize for Invention by the Invest-and Structure-Bank of Rhineland-Palatinate, the Vikram Sarahbai Memorial Oration Award, the most important honour of the Society of Nuclear Medicine in India and finally with the Hevesy Medal Award. In his scientific projects he worked on a variety of radionuclides, e.g., ^211^At, ^99m^Tc, ^86^Y, ^90^Y, ^44^Sc, ^68^Ga, ^188^Re, ^18^F, etc. and their production, purification, physicochemical properties and radiolabeling, up to preclinical and clinical studies. The production pathway for carrier-free ^177^Lu via the ^176^Yb-route, which since than became the most important therapeutic isotope and the cation exchange-based purification of the ^68^Ge/^68^Ga-generator eluate, which is nowadays the most important post-processing for this generator worldwide, were important milestones in his scientific career. His impact is not deniable and as such, we are happy to be a part of this dedicated Research Topic, which consists of six research articles/reviews celebrating the long-term outstanding achievements and contributions of Prof. Dr. Frank Rösch.

In the following, we want to summarize in short the different contributions to this Research Topic.

Zippel et al. presents an academically-updated overview of the medical cyclotrons operated for the production of radiopharmaceuticals and their use in Nuclear Medicine in German speaking countries. It identifies 42 cyclotrons in operation, mostly in Germany and underscore cyclotrons' pivotal role in nuclear medicine's growth, necessitating ongoing infrastructure development and industry-academia collaboration to meet rising demand.

Lopes van den Broek et al. (Nov. 2022) focusses on the development and evaluation of an ^18^F-labeled nanobody which targets the SARS-CoV-2 spike protein with the aim of visualizing the virus itself. The nanobodies are decorated with *trans*-cyclooctenes (TCOs), which enables ^18^F-labeling under mild conditions through the use of the tetrazine ligation.

Greifenstein et al. describes squaric acid-based bisphosphonate as theranostics for bone metastasis. Their initial findings reveal that these compounds exhibit remarkable affinity for bone tissue, resulting in high uptake and specificity within bone structures which makes these compounds highly promising candidates for improving the precision and accuracy of bone metastasis imaging and diagnosis.

Grus et al. describes a dual-targeting approach by combining a PSMA inhibitor, facilitating specific cancer cell recognition, with a bisphosphonate known for its bone-targeting properties. Their compound, DOTA-L-Lys(SA.Pam)-PSMA-617 exhibits a robust affinity for both PSMA receptors and bone tissue, indicating its potential as an effective theranostic agent for prostate cancer.

Bajwa et al. shows a radiolabeled iron oxide nanoparticles decorated with PSMA and GRPR targeting ligands. These nanoparticles, tagged with ^99m^Tc, demonstrated remarkable stability and an exceptional safety profile in prostate cancer cell lines.

Finally, Lopes van den Broek et al. (Sept. 2022) explores a pretargeted imaging approach, utilizing an amyloid β specific antibody. This antibody can bioorthogonally be labeled with short-lived radionuclides, through the tetrazine ligation. Three AD mouse models, 5xFAD, APP/PS1, and tg-ArcSwe, were evaluated for their suitability in pretargeted imaging beyond the blood-brain barrier.

